# Genetic loci associated with circulating levels of very long-chain saturated fatty acids[S]

**DOI:** 10.1194/jlr.M052456

**Published:** 2015-01

**Authors:** Rozenn N. Lemaitre, Irena B. King, Edmond K. Kabagambe, Jason H. Y. Wu, Barbara McKnight, Ani Manichaikul, Weihua Guan, Qi Sun, Daniel I. Chasman, Millennia Foy, Lu Wang, Jingwen Zhu, David S. Siscovick, Michael Y. Tsai, Donna K. Arnett, Bruce M. Psaty, Luc Djousse, Yii-Der I. Chen, Weihong Tang, Lu-Chen Weng, Hongyu Wu, Majken K. Jensen, Audrey Y. Chu, David R. Jacobs, Stephen S. Rich, Dariush Mozaffarian, Lyn Steffen, Eric B. Rimm, Frank B. Hu, Paul M. Ridker, Myriam Fornage, Yechiel Friedlander

**Affiliations:** * Cardiovascular Health Research Unit, Department of Medicine, University of Washington, Seattle, WA; ‖ Department of Biostatistics, School of Public Health, University of Washington, Seattle, WA; $$ Departments of Epidemiology University of Washington, Seattle, WA; ‡‡‡ Health Services, University of Washington, Seattle, WA; † Department of Internal Medicine, University of New Mexico, Albuquerque, NM; ‡ Division of Epidemiology, Department of Medicine, Vanderbilt University Medical Center, Nashville, TN; § The George Institute for Global Health, University of Sydney, Sydney, Australia; # Center for Public Health Genomics University of Virginia, Charlottesville, VA; $ Department of Public Health Sciences, Division of Biostatistics, University of Virginia, Charlottesville, VA; ** Division of Biostatistics, School of Public Health, University of Minnesota, Minneapolis, MN; *** Department of Laboratory Medicine and Pathology, University of Minnesota, Minneapolis, MN; **** Division of Epidemiology and Community Health, School of Public Health, University of Minnesota, Minneapolis, MN; †† Channing Division of Network Medicine, Department of Medicine, Brigham and Women’s Hospital and Harvard Medical School, Boston, MA; §§ Division of Preventive Medicine, Department of Medicine, Brigham and Women’s Hospital and Harvard Medical School, Boston, MA; ‖‖‖ Department of Medicine, Brigham and Women’s Hospital and Harvard Medical School, Boston, MA; ‡‡‡‡ Division of Cardiovascular Medicine, Department of Medicine, Brigham and Women’s Hospital and Harvard Medical School, Boston, MA; ‡‡ Departments of Nutrition Harvard School of Public Health, Boston, MA; †††† Epidemiology, Harvard School of Public Health, Boston, MA; ‖‖ Institute of Molecular Medicine University of Texas Health Sciences Center-Houston, Houston, TX; #### Division of Epidemiology, Human Genetics, and Environmental Sciences, University of Texas Health Sciences Center-Houston, Houston, TX; ## Key Laboratory of Nutrition and Metabolism Institute for Nutritional Sciences, Shanghai Institutes for Biological Sciences, Chinese Academy of Sciences and Graduate University of the Chinese Academy of Sciences, Shanghai, People’s Republic of China; ††† Department of Epidemiology, University of Alabama at Birmingham, Birmingham, AL; §§§ Group Health Research Institute, Group Health Cooperative, Seattle, WA; ### Boston Veterans Healthcare System, Boston, MA; $$$ Medical Genetics Research Institute, Cedars-Sinai Medical Center, Los Angeles, CA; §§§§ Friedman School of Nutrition Science and Policy, Tufts University, Boston, MA; ‖‖‖‖ Division of Cardiovascular Medicine, Department of Medicine, Brigham and Women’s Hospital, Boston, MA; $$$$ Braun School of Public Health, Hebrew University-Hadassah Medical Center, Jerusalem, Israel

**Keywords:** arachidic acid, behenic acid, lignoceric acid, sphingolipids

## Abstract

Very long-chain saturated fatty acids (VLSFAs) are saturated fatty acids with 20 or more carbons. In contrast to the more abundant saturated fatty acids, such as palmitic acid, there is growing evidence that circulating VLSFAs may have beneficial biological properties. Whether genetic factors influence circulating levels of VLSFAs is not known. We investigated the association of common genetic variation with plasma phospholipid/erythrocyte levels of three VLSFAs by performing genome-wide association studies in seven population-based cohorts comprising 10,129 subjects of European ancestry. We observed associations of circulating VLSFA concentrations with common variants in two genes, serine palmitoyl-transferase long-chain base subunit 3 (*SPTLC3*), a gene involved in the rate-limiting step of de novo sphingolipid synthesis, and ceramide synthase 4 (*CERS4*). The *SPTLC3* variant at rs680379 was associated with higher arachidic acid (20:0 , *P* = 5.81 × 10^−13^). The *CERS4* variant at rs2100944 was associated with higher levels of 20:0 (*P* = 2.65 × 10^−40^) and in analyses that adjusted for 20:0, with lower levels of behenic acid (*P* = 4.22 × 10^−26^) and lignoceric acid (*P* = 3.20 × 10^−21^). These novel associations suggest an inter-relationship of circulating VLSFAs and sphingolipid synthesis.

Very long-chain saturated fatty acids (VLSFAs) are saturated fatty acids with 20 or more carbons, including arachidic acid [20:0 (20 carbons and 0 double bonds)], behenic acid (22:0), and lignoceric acid (24:0). Well-known as markers of peroxisomal disorders (1), VLSFAs also play important roles in normal physiology (2). VLSFAs are components of sphingolipids (3), such as sphingomyelin and ceramides, and impart specific biological activities to the sphingolipids (4). For example, sphingolipids with VLSFAs promote the formation of lipid microdomains in plasma membranes (5). In addition, ceramides with VLSFAs show biological activities opposite to ceramides with palmitic acid (16:0), a long-chain saturated fatty acid (6).

Recent studies in human populations suggest beneficial effects of circulating levels of VLSFAs (7–9). We previously reported that higher levels of VLSFAs in plasma phospholipids were associated with a lower risk of incident atrial fibrillation in the Cardiovascular Health Study (CHS) (7). More recently, we showed that higher levels of erythrocyte VLSFAs were associated with lower risk of incident sudden cardiac arrest in a population-based case-control study, where VLSFAs were measured in blood samples collected at the time of cardiac arrest (8). In addition, a recent report from the European Prospective Investigation into Cancer and Nutrition (EPIC)-InterAct case-cohort study showed an association of higher levels of plasma phospholipid VLSFAs with a lower risk of incident type 2 diabetes (9). In contrast, higher levels of circulating 16:0 are associated with higher risks of atrial fibrillation (7), sudden cardiac arrest (8), and diabetes (9). These studies suggest a need to differentiate between saturated fatty acids of different lengths and a need to study the determinants of circulating VLSFAs.

The fatty acids 20:0, 22:0, and 24:0 are primarily found in peanuts, peanut oil, cashews, macadamia nuts, canola oil, and in trace amounts in other nuts and oils (10). Consumption of VLSFAs appears to raise circulating levels of VLSFAs. (11, 12). Dietary supplementation with macadamia nuts at 15% of total energy raised plasma levels of 20:0 and 22:0 (11); and in another small trial, addition of peanut butter to the regular diet raised plasma levels of 22:0 and 24:0 (12). Apart from dietary sources, VLSFAs are also formed endogenously by elongation of 16:0 (13). In mammalian systems, three elongases (elovls) are known to contribute to the production of VLSFAs: elovl6 produces stearic acid (18:0) from 16:0; elovl1, elovl3, and elovl7 all have the ability to elongate 18:0 to 20:0 and 20:0 to 22:0; and elovl1 and elovl3 elongate 22:0 to 24:0 (13, 14). While diets, such as high-carbohydrate/low-fat diets, and alcohol consumption are known to influence de novo synthesis of 16:0 (15–17), dietary and metabolic factors that may regulate the elongation of 16:0 to VLSFAs are not well-known. In the current study, we investigated genetic factors that may influence circulating levels of VLSFAs.

We have previously found evidence of high heritability of erythrocyte levels of 24:0 in the Kibbutzim Family Study (18). To identify common genetic variants that might influence levels of circulating 20:0, 22:0, and 24:0, we conducted a meta-analysis of genome-wide association studies (GWASs) of plasma phospholipid/erythrocyte VLSFAs in 10,129 participants of European ancestry in seven cohort studies, as part of the Cohorts for Heart and Aging Research in Genomic Epidemiology (CHARGE) Consortium.

## SUBJECTS AND METHODS

### Ethics statement

Each cohort that participated in this study secured approval from their respective institutional review boards, and all participants provided written informed consent in accordance with the Declaration of Helsinki.

### Study samples

We conducted a meta-analysis of data from seven cohorts comprising 10,129 individuals of European ancestry (**Table 1**). Participating cohorts included the Atherosclerosis Risk in Communities (ARIC) study, the Coronary Artery Risk Development in Young Adults (CARDIA) study, the CHS, the Health Professionals Follow-up Study (HPFS), the Multi-Ethnic Study of Atherosclerosis (MESA), the Nurses’ Health Study (NHS), and the Women’s Genome Health Study (WGHS). Only data from participants of European ancestry were included in the current analysis. Descriptions of each participating cohort are summarized in the supplementary text.

**TABLE 1.** CHARGE cohorts description

					Fatty Acid Concentration (% of total fatty acids)		
Cohort	N	Age (years)	Men (%)	Fatty Acid Measured In	20:0	22:0	24:0
ARIC	3,269	53.8 (5.6)	48.7	Plasma phospholipids	0.19 (0.04)	0.57 (0.15)	0.49 (0.15)
CARDIA	1,507	45.6 (3.3)	46.7	Plasma phospholipids	0.23 (0.08)	0.61 (0.28)	NA
MESA	707	61.6 (10.4)	46.8	Plasma phospholipids	0.23 (0.09)	0.48 (0.27)	NA
CHS	2,404	75.0 (5.1)	38.4	Plasma phospholipids	0.49 (0.08)	1.65 (0.31)	1.38 (0.28)
HPFS	1,295	63.6 (8.6)	100	Erythrocytes	0.38 (0.05)	1.58 (0.27)	3.91 (0.80)
NHS	295	60.3 (6.1)	0	Erythrocytes	0.39 (0.09)	1.48 (0.30)	2.79 (0.84)
WGHS	652	54.4 (6.5)	0	Erythrocytes	0.25 (0.12)	0.87 (0.26)	2.06 (0.54)

Values in the table are mean (SD) except where specified otherwise. 24:0 was not measured in CARDIA and MESA. NA, not available.

### Fatty acid measurements

Fatty acids were measured in plasma phospholipids in ARIC, CARDIA, CHS, and MESA, and in erythrocyte membranes in HPFS, NHS, and WGHS. Details of the fatty acid measurements are provided in the supplementary text. While the fatty acids 20:0 and 22:0 were measured in all cohorts, 24:0 was not available in CARDIA and MESA. Levels of 20:0, 22:0, and 24:0 were expressed as percent of total fatty acids.

### Imputation and statistical analysis

Genotyping was performed separately in each cohort using high-density SNP marker platforms [ARIC, CARDIA, MESA, HPFS, and NHS (Affymetrix 6.0); CHS (Illumina 370); HPFS, NHS, and WGHS (Illumina HumanHap 300 DuoPlus)]. Samples with call rates below 95% (ARIC, CARDIA, MESA), 97% (CHS), or 98% (HPFS, NHS, WGHS) at genotyped markers were excluded. Genotypes were imputed to approximately 2.5 million HapMap SNPs by using either MaCH (19) (ARIC, NPHS, NHS, and WGHS), BIMBAM (20) (CHS), BEAGLE (21) (CARDIA), or IMPUTE (22) (MESA). SNPs for which testing Hardy-Weinberg equilibrium resulted in *P <* 10^−4^ (HPFS, NHS), *P <* 10^−5^ (CHS), or *P* < 10^−6^ (ARIC, WGHS) were excluded from imputation. SNPs with minor allele frequency (MAF) ≤1% or effective degree of freedom (2 × MAF × sample size × observed divided by expected variance for imputed allele dosage) ≤50 were excluded from the meta-analyses. Additional details on genotyping and imputation per cohort are provided in the supplementary text.

Association analysis between genotype and each fatty acid was performed separately within each study cohort according to a prespecified analysis plan. All studies conducted linear regression analysis measuring the additive effect of the number of effect alleles, or equivalently the imputed number of effect alleles for imputed genotypes. In absence of a known model, we chose the additive model a priori because it has good power for all “monotone” models, including recessive and dominant (23). The analyses used robust standard errors and were adjusted for age, sex, site of recruitment where appropriate, and where needed, principal components to account for possible population genetic substructure.

### Meta-analysis

Because circulating levels of the fatty acids of interest differed across the cohorts (Table 1) and because combining effect sizes requires that the trait is measured on exactly the same scale in each study, we performed a *z* score-based meta-analysis of each fatty acid, as implemented in METAL (Developed by Goncalo Abecasis at the University of Michigan). Genomic control correction was applied to each study prior to the meta-analysis and correction factors ranged from 0.96 to 1.03 (20:0), 1.00 to 1.03 (22:0), and 0.99 to 1.03 (24:0). *P* values less than 5 × 10^−8^ were considered statistically significant.

Our approach, to include all the studies in a meta-analysis, has more power than splitting the studies into a discovery sample and a replication sample (24).

In sensitivity analyses, we performed fixed-effects meta-analyses in which studies were stratified according to whether fatty acids were measured in plasma phospholipids or erythrocytes. The fixed effect meta-analyses were conducted for the associations of three SNPs, discovered with the *z* score-based meta-analysis, and using standard deviation units for the fatty acids to minimize study to study differences in the measurement of the VLSFAs. We tested for a difference between plasma phospholipids and erythrocytes using fixed effect meta-analyses regression (25).

## RESULTS

Table 1 presents demographic characteristics and mean levels of circulating 20:0, 22:0, and 24:0 for the study sample of up to 10,129 subjects of European ancestry in the ARIC, CARDIA, CHS, HPFS, MESA, NHS, and WGHS cohorts. The cohorts differed by age (mean age ranged from 45.6 to 75.0 years) and gender (percent of men ranged from 0 to 100%). Levels of VLSFAs varied with the fatty acid compartment measured (erythrocyte vs. plasma phospholipid) and by cohort. The higher levels of VLSFAs in the CHS cohort, compared with other cohorts using plasma phospholipids, may be due to a higher recovery of these hydrophobic fatty acids with the method used in the CHS for the extraction of fatty acid methyl esters (details in the supplementary text).

**Fig. 1.**
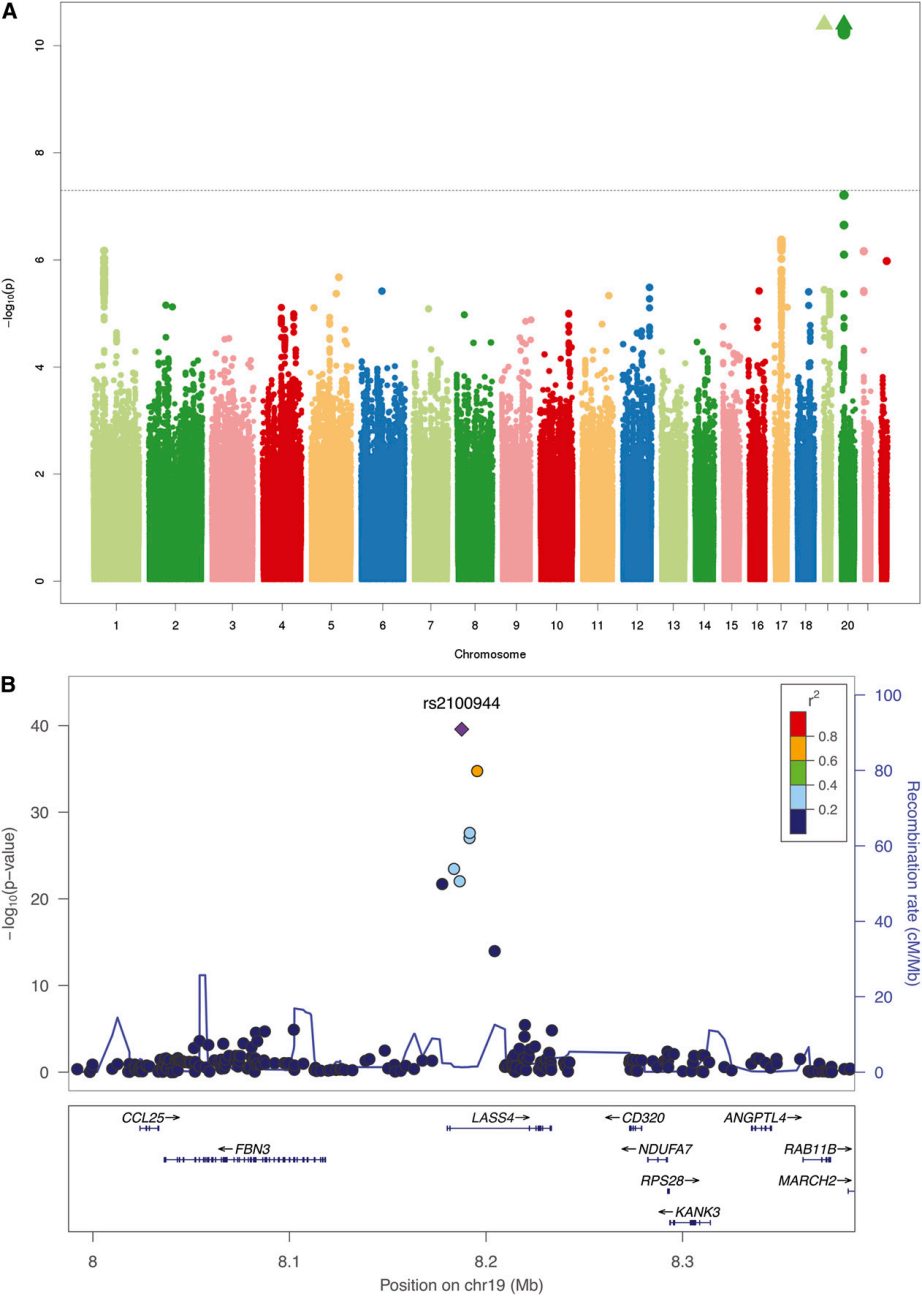

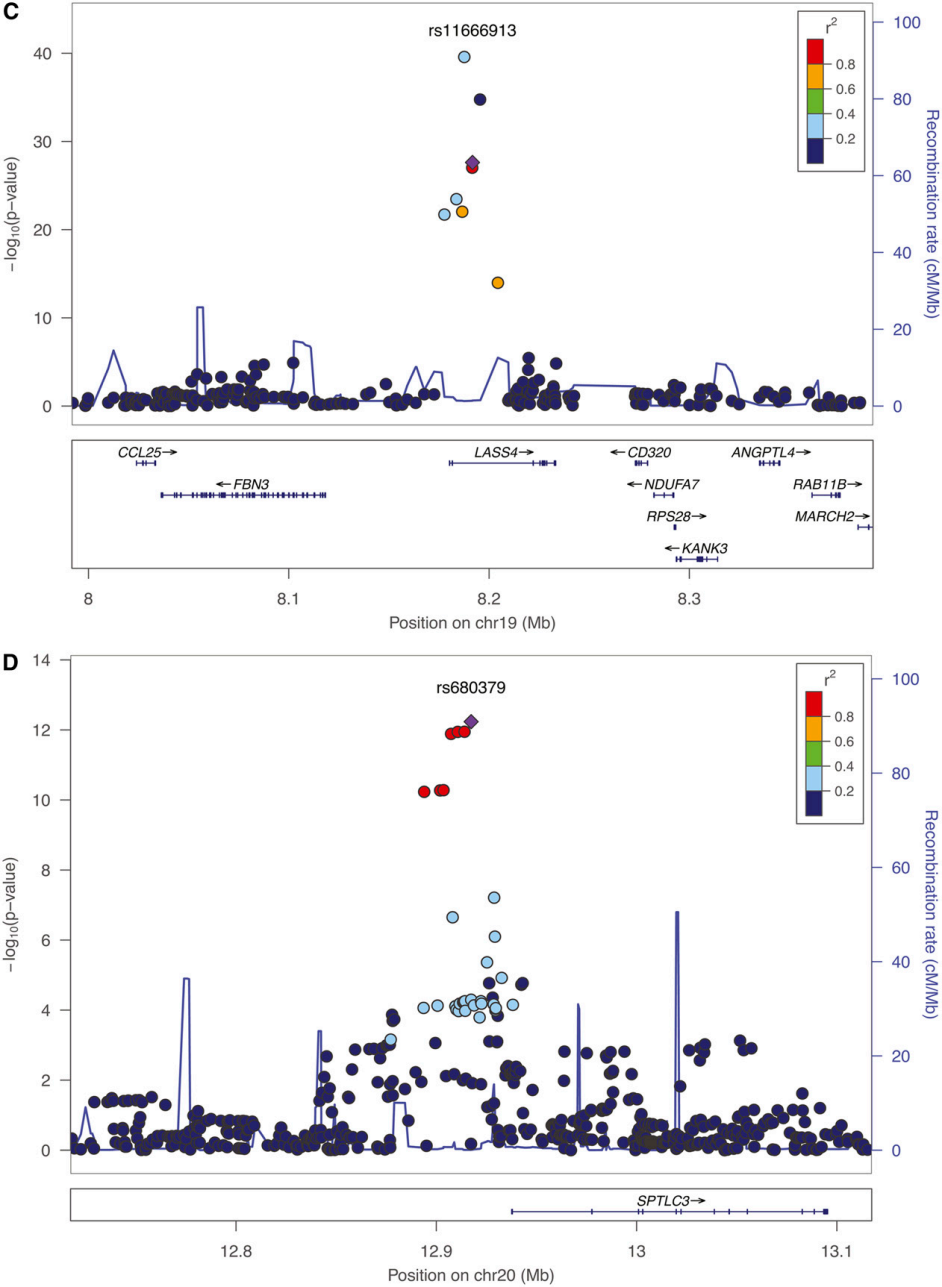
A: Meta-analysis of genome-wide associations with 20:0. Associations were graphed by chromosome position and –log_10_ (*P* value) up to *P* values of 10^−10^. Triangles indicate additional SNPs with *P* values less than 10^−10^. B–D: SNP association plots for 20:0-associated regions. Genome-wide association significance level is plotted against the *y* axis as –log_10_ (*P* value). Genetic coordinates are as per National Cancer Institute build 36. B: *CERS4* (*LASS4*) region. LD is indicated by color scale in relationship to marker rs2100944. C: *CERS4* (*LASS4*) region. LD is indicated by color scale in relationship to marker rs11666913. D: *SPTLC3* region. LD is indicated by color scale in relationship to marker rs680379. The color scheme in (B–D) is red for *r*^2^ ≥ 0.8, orange for *r*^2^ ≥ 0.6 and <0.8, green for *r*^2^ ≥ 0.4 and <0.6, blue for *r*^2^ ≥ 0.2 and <0.4, purple for *r*^2^ < 0.2.

The meta-analysis of GWAS results revealed two genetic loci associated with circulating levels of 20:0 at genome-wide significance (**Table 2**, **Fig. 1**, and supplementary Tables 1, 2). The loci are illustrated in the association plots in Fig. 1B–D. Eight SNPs on chromosome 19p13.2, in the ceramide synthase 4 (*CERS4*) gene, were associated with levels of 20:0. The variant allele of the most significant SNP (rs2100944, *P* = 2.65 × 10^−40^) was negatively associated with levels of 20:0, while another variant allele (rs11666913, *P* = 2.43 × 10^−28^) in modest linkage disequilibrium (LD) with rs2100944 (*r*^2^ = 0.22) was positively associated with levels of 20:0. The other locus, on chromosome 20p12.1, contained the serine palmitoyl-transferase long-chain base subunit 3 (*SPTLC3*) gene. Variant alleles at seven highly correlated SNPs in this locus were associated with higher levels of 20:0 at genome-wide levels of significance, and the most significant variant allele was at rs680379 (*P* = 5.81 × 10^−13^) (Table 2). The directions of associations of the variant alleles were consistent in all cohorts where results for these variants were available (supplementary Table 1).

**TABLE 2.** Loci associated with SNP markers with P values < 5.0 × 10^−8^

			Most Significant SNP			
Fatty Acid and Chromosome (Main Genes)	Analysis	Number of Significant SNPs	rs Number	Minor allele/Other allele	MAF	*P*
20:0						
19 (*CERS4*)	Main results	8	rs2100944	G/A	0.224	2.65 × 10^−40^
			rs11666913	C/G	0.407	2.43 × 10^−28^
20 (*SPTLC3*)	Main results	7	rs680379	A/G	0.378	5.81 × 10^−13^
22:0						
19 (*CERS4*)	Main results	0				
	With adjustment for 20:0	8	rs2100944	G/A	0.224	4.22 × 10^−26^
			rs11666913	C/G	0.407	3.27 × 10^−16^
24:0						
19 (*CERS4*)	Main results	0				
	With adjustment for 20:0	8	rs2100944	G/A	0.224	3.20 × 10^−21^
			rs11666913	C/G	0.407	1.27 × 10^−17^

No genome-wide significant associations were observed with circulating levels of 22:0 and 24:0 (supplementary Figs. 1A, 2A). However, levels of 20:0, 22:0, and 24:0 are positively correlated, with correlation coefficients ranging from 0.63 to 0.89 for 20:0-22:0, from 0.48 to 0.70 for 20:0-24:0, and from 0.79 to 0.90 for 22:0-24:0 in the study cohorts. When two fatty acids are positively correlated but exhibit genetic associations in opposite directions, it is possible to increase the power of discovery efforts by adjusting the association between SNPs and one fatty acid trait for the other fatty acid (26). We therefore conducted GWASs of 22:0 and 24:0 with adjustment for 20:0 (supplementary Figs. 1B, 2B). In these analyses, the same eight SNPs in *CERS4* that were genome-wide significant in the GWAS of 20:0, became genome-wide significant in the GWASs of 22:0 and 24:0, and all associations were in the opposite directions to those with 20:0 (Table 2; supplementary Tables 3, 4). The variant allele of the SNP most associated with 20:0 (rs2100944) was positively associated with 22:0 (*P* = 4.22 × 10^−26^) and with 24:0 (*P* = 3.20 × 10^−21^); and the variant allele of rs11666913 was negatively associated with 22:0 (*P* = 3.27 × 10^−16^) and 24:0 (*P* = 1.27 × 10^−17^).

In sensitivity analyses, we repeated the meta-analysis of the associations of rs2100944, rs11666913, and rs680379 with VLSFAs to compare results in plasma phospholipids and erythrocytes. We saw evidence for smaller effect sizes in erythrocytes than in plasma phospholipids in the associations of rs2100944 and rs11666913 with each VLSFA, but not in the association of rs680379 with 20:0 (supplementary Tables 5, 6).

## DISCUSSION

**Fig. 2.**
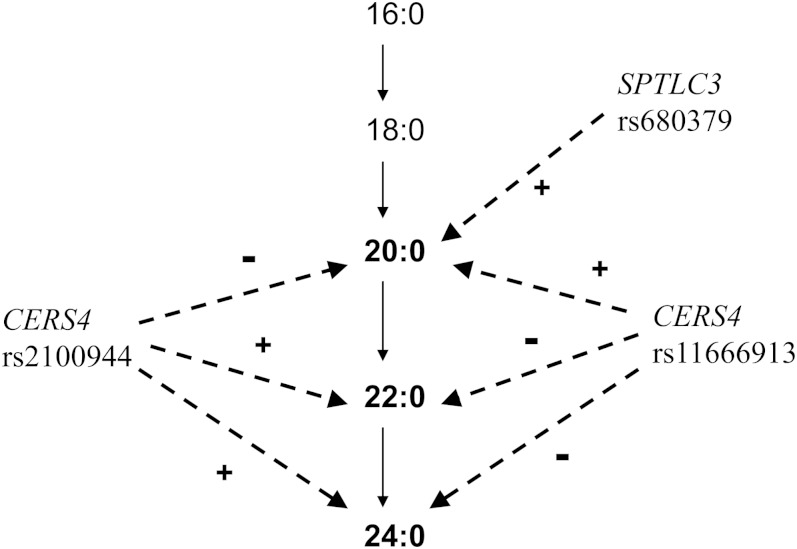
Metabolic pathway from 16:0 to 24:0 and summary of genome-wide associations in pathway. The fatty acids indicated in bold were examined in this study. The genome-wide significant associations of 20:0, 22:0, and 24:0 with three variant alleles in two genes are shown with dashed arrows. + and − signs indicate the direction of the associations.

In this large meta-analysis, we report for the first time that variation in two genes involved in sphingolipid biosynthesis is significantly associated with circulating levels of VLSFAs. Specifically, levels of the fatty acid 20:0 were associated with common variation in *SPTLC3* and in *CERS4*. After adjustment for levels of 20:0, levels of 22:0 and 24:0 were also associated with variation in *CERS4*, but in a direction opposite to that of 20:0 (**Fig. 2**).

**Fig. 3.**
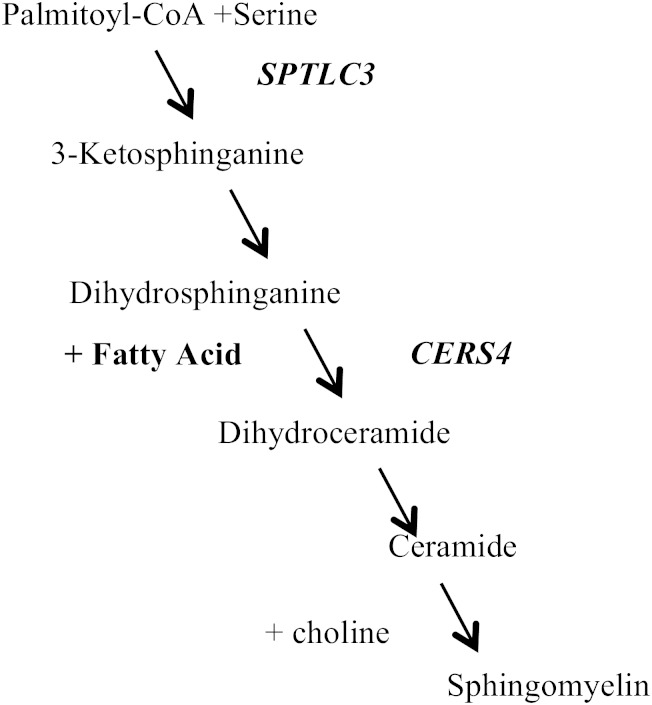
De novo biosynthesis of ceramides and sphingomyelins. The figure shows the pathway location of the two genes associated with levels of VLSFAs. *SPTLC3* produces 3-ketosphinganine (sphingoid backbone) in the first rate-limiting step of the pathway. *CERS4* introduces a VLSFA into the backbone to produce dihydroceramide, the precursor of ceramide and sphingomyelin.

*SPTLC3* codes for the enzyme of the first step of sphingolipid de novo biosynthesis (27) (**Fig. 3**). The enzyme condenses 16:0 or myristic acid with the amino acid serine resulting in the “sphingoid” backbone of all sphingolipids. This step is a rate-limiting step that influences the flow through the de novo synthesis pathway. VLSFAs are used at a downstream step to form ceramides (Fig. 3), and ceramides can be metabolized into sphingomyelin, a phospholipid. Interestingly, the SNP in *SPTLC3* that was most strongly associated with circulating levels of 20:0, rs680379, was also associated with circulating levels of ceramides containing 22:0 and ceramides containing 24:0 in the European Special Population Research Network (EUROSPAN) (28). These observations suggest that plasma phospholipid/erythrocyte levels of 20:0 may reflect sphingomyelin produced by de novo synthesis.

*CERS4* encodes for a ceramide synthase, an enzyme that introduces a fatty acid, such as 20:0, into the sphingoid backbone (generated by sptlc1, 2, or 3), thereby producing dihydroceramide, a precursor of ceramides and sphingomyelins (Fig. 3). Ceramide synthases also produce ceramide from sphingosine that is recycled from the breakdown of sphingomyelin and other sphingolipids, although the relative contribution of the de novo synthesis and recycling pathways to ceramide production is not well-understood (29). Humans have six genes encoding ceramide synthases with different specificity for saturated fatty acids of different lengths (30). *CERS4* uses preferentially 20:0 or 18:0 to produce ceramide containing 20:0 or ceramide with 18:0. Several of genetic loci most closely associated with circulating 20:0 have also been found associated with circulating ceramides containing VLSFAs in the GWAS of plasma sphingolipids in the EUROSPAN (28). In particular, rs11666866 (a *CERS4* SNP also in our top hits, with *r*^2^ of 0.97 with rs11666913) was associated with higher levels of plasma ceramide containing 20:0, mirroring the association of rs11666866 and rs11666913 with higher levels of circulating 20:0 that we observed. Likewise, rs1466448 (another *CERS4* SNP in our top hits, with an *r*^2^ of 0.69 with rs2100944) was associated with lower levels of ceramide with 20:0 in EUROSPAN, again similar to the association of rs1466448 and rs2100944 with lower levels of circulating 20:0 in our study. The associations of *CERS4* variants, in the same direction, with both circulating levels of ceramides containing 20:0 and overall plasma phospholipid/erythrocyte levels of 20:0, which include sphingomyelin in addition to phosphoglycerolipids, suggest that incorporation into sphingolipids is an important metabolic fate of 20:0. This observation is generally consistent with the known fatty acid composition of sphingolipids (31, 32).

We observed opposite association of *CERS4* variants with 20:0 versus 22:0 and 24:0 (Fig. 2), with a set of variants associated with higher 20:0 and lower 22:0 and 24:0, and another set of variants associated with lower 20:0 and higher 22:0 and 24:0. An intriguing possibility is that genetic variation in *CERS4* might alter the specificity of the enzyme, with some variants favoring 20:0 over 22:0 or 24:0 and other variants favoring 22:0 or 24:0 over 20:0. Our results highlight the need to identify the functional allele(s) in these loci that may lead to alterations in VLSFA levels.

We recently reported genome-wide associations with the saturated fatty acids 16:0 and 18:0 (33). None of the reported findings for 16:0 and 18:0 overlapped with the genome-wide association findings with VLSFAs in this study. In particular, 18:0 was associated with variation in *LPGAT1*, an enzyme involved in phosphatidylglycerol synthesis, whereas VLSFAs were associated with genes involved in sphingolipid synthesis in the current study. These observations further highlight likely differences in the metabolism of saturated fatty acids of different lengths, and the need for more targeted research of individual saturated fatty acids to understand their roles in health and disease.

Little is known of the biological activities of VLSFAs, yet emerging evidence suggests possible beneficial properties. We previously reported an inverse association of plasma phospholipid VLSFAs with risk of incident atrial fibrillation (7), and more recently we found an inverse association of erythrocyte VLSFAs with risk of sudden cardiac arrest (8). In addition, Forouhi et al. (9) reported an inverse association of plasma phospholipid VLSFAs with risk of incident type 2 diabetes. Interestingly, the VLSFAs that are incorporated into ceramides also impart protective activities (6). Ceramides are best known for promoting apoptosis (34, 35); however, the saturated fatty acid that is N-acylated to the sphingoid backbone appears to modify ceramide biological activities (6). For example, in the worm *Caenorhabditis elegans*, ceramide with 16:0 promotes apoptosis (36), but ceramides with 20:0 or 22:0 are protective against hypoxia-induced apoptosis (37). Similarly, in a mouse model of lipotoxic cardiomyopathy, treatment with myriocin lowers total ceramides and enhances cardiomyocyte survival (34); however, in genetically engineered mice with lower levels of ceramides with 18:0, 20:0, and 24:0, the heart shows more (not less) apoptosis (38). Involvement of VLSFAs in sphingolipid metabolism, highlighted in the present study findings, suggests sphingolipids may mediate the reported VLSFA associations. Drug therapies exist that influence global levels of sphingolipids [e.g., Fenretinide (39)]. If sphingolipids with specific fatty acids are shown to be protective of arrhythmias and diabetes, it may spur the development of more selective drug therapies, and promote drug and dietary trials to alter sphingolipid composition and disease risk.

Our study has several strengths that include the use of samples from population-based cohort studies to enhance generalizability of findings; the measurement of VLSFAs in plasma or erythrocyte phospholipids, objective biomarkers of tissue membrane phospholipids; and the large sample size that allowed detection of genome-wide significant associations. Additionally, we took advantage of correlations between the VLSFAs to increase the power of detecting associations with 22:0 and 24:0.

Potential limitations should also be considered. The top SNPs may tag less common SNPs that are responsible for the observed associations, and resequencing of the loci may be needed to identify potentially causal variants. The VLSFAs were measured in plasma phospholipids in some cohorts and erythrocytes in the others, and the levels varied with the methodology that was used. Although we addressed this limitation by using *z* scores in our meta-analysis, residual heterogeneity remained possible. It is reassuring to see that the directions of the associations for the top SNPs were consistent in all the cohorts. The study only included participants of European ancestry and further studies are needed to confirm the associations in other ethnic groups.

In conclusion, we identified novel associations of common variants in two sphingolipid genes with circulating levels of VLSFAs. These novel associations suggest an inter-relationship of circulating VLSFAs and sphingolipid endogenous synthesis.

## Supplementary Material

Supplemental Data

## References

[1] Moser A. B. Kreiter N. Bezman L. Lu S. Raymond G. V. Naidu S. Moser H. W. 1999 Plasma very long chain fatty acids in 3,000 peroxisome disease patients and 29,000 controls. Ann. Neurol. 45: 100–110. 9894883 10.1002/1531-8249(199901)45:1<100::aid-art16>3.0.co;2-u

[2] Kihara A. 2012 Very long-chain fatty acids: elongation, physiology and related disorders. J. Biochem. 152: 387–395. 22984005 10.1093/jb/mvs105

[3] Quehenberger O. Armando A. M. Brown A. H. Milne S. B. Myers D. S. Merrill A. H. Bandyopadhyay S. Jones K. N. Kelly S. Shaner R. L. 2010 Lipidomics reveals a remarkable diversity of lipids in human plasma. J. Lipid Res. 51: 3299–3305. 20671299 10.1194/jlr.M009449 PMC2952570

[4] Sassa T. Suto S. Okayasu Y. Kihara A. 2012 A shift in sphingolipid composition from C24 to C16 increases susceptibility to apoptosis in HeLa cells. Biochim. Biophys. Acta. 1821: 1031–1037. 22579584 10.1016/j.bbalip.2012.04.008

[5] Iwabuchi K. Nakayama H. Iwahara C. Takamori K. 2010 Significance of glycosphingolipid fatty acid chain length on membrane microdomain-mediated signal transduction. FEBS Lett. 584: 1642–1652. 19852959 10.1016/j.febslet.2009.10.043

[6] Grösch S. Schiffmann S. Geisslinger G. 2012 Chain length-specific properties of ceramides. Prog. Lipid Res. 51: 50–62. 22133871 10.1016/j.plipres.2011.11.001

[7] Fretts A. M. Mozaffarian D. Siscovick D. S. Djousse L. Heckbert S. R. King I. B. McKnight B. Sitlani C. Sacks F. M. Song X. 2014 Plasma phospholipid saturated fatty acids and incident atrial fibrillation: the Cardiovascular Health Study. J. Am. Heart Assoc. 3: e000889. 24970268 10.1161/JAHA.114.000889 PMC4309088

[8] Lemaitre R. N. King I. B. Rice K. McKnight B. Sotoodehnia N. Rea T. D. Johnson C. O. Raghunathan T. E. Cobb L. A. Mozaffarian D. 2014 Erythrocyte very long-chain saturated fatty acids associated with lower risk of incident sudden cardiac arrest. Prostaglandins Leukot. Essent. Fatty Acids. 91: 149–153. 25107579 10.1016/j.plefa.2014.07.010 PMC4156887

[9] Forouhi N. G. Koulman A. Sharp S. J. Imamura F. Kröger J. Schulze M. B. Crowe F. L. Huerta J. M. Guevara M. Beulens J. W. J. 2014 Differences in the prospective association between individual plasma phospholipid saturated fatty acids and incident type 2 diabetes: the EPIC-InterAct case-cohort study. Lancet Diabetes Endocrinol. 2: 810–818. 25107467 10.1016/S2213-8587(14)70146-9 PMC4196248

[10] US Department of Agriculture Agricultural Research Service. 2014 USDA National Nutrient Database for Standard Reference, Release 27. Accessed 10/1/2014 at http://www.ars.usda.gov/ba/bhnrc/ndl.

[11] Garg M. L. Blake R. J. Wills R. B. 2003 Macadamia nut consumption lowers plasma total and LDL cholesterol levels in hypercholesterolemic men. J. Nutr. 133: 1060–1063. 12672919 10.1093/jn/133.4.1060

[12] Lam C. Wong D. Cederbaum S. Lim B. Qu Y. 2012 Peanut consumption increases levels of plasma very long chain fatty acids in humans. Mol. Genet. Metab. 107: 620–622. 22864056 10.1016/j.ymgme.2012.07.015

[13] Jakobsson A. Westerberg R. Jacobsson A. 2006 Fatty acid elongases in mammals: their regulation and roles in metabolism. Prog. Lipid Res. 45: 237–249. 16564093 10.1016/j.plipres.2006.01.004

[14] Guillou H. Zadravec D. Martin P. G. Jacobsson A. 2010 The key roles of elongases and desaturases in mammalian fatty acid metabolism: Insights from transgenic mice. Prog. Lipid Res. 49: 186–199. 20018209 10.1016/j.plipres.2009.12.002

[15] Knopp R. H. Retzlaff B. Walden C. Fish B. Buck B. McCann B. 2000 One-year effects of increasingly fat-restricted, carbohydrate-enriched diets on lipoprotein levels in free-living subjects. Proc. Soc. Exp. Biol. Med. 225: 191–199. 11082213 10.1046/j.1525-1373.2000.22524.x

[16] Hudgins L. C. Hellerstein M. Seidman C. Neese R. Diakun J. Hirsch J. 1996 Human fatty acid synthesis is stimulated by a eucaloric low fat, high carbohydrate diet. J. Clin. Invest. 97: 2081–2091. 8621798 10.1172/JCI118645 PMC507283

[17] King I. B. Lemaitre R. N. Kestin M. 2006 Effect of a low-fat diet on fatty acid composition in red cells, plasma phospholipids, and cholesterol esters: investigation of a biomarker of total fat intake. Am. J. Clin. Nutr. 83: 227–236. 16469979 10.1093/ajcn/83.2.227

[18] Lemaitre R. N. Siscovick D. S. Berry E. M. Kark J. D. Friedlander Y. 2008 Familial aggregation of red blood cell membrane fatty acid composition: the Kibbutzim Family Study. Metabolism. 57: 662–668. 18442630 10.1016/j.metabol.2007.12.011

[19] Li Y. Willer C. J. Ding J. Scheet P. Abecasis G. R. 2010 MaCH: using sequence and genotype data to estimate haplotypes and unobserved genotypes. Genet. Epidemiol. 34: 816–834. 21058334 10.1002/gepi.20533 PMC3175618

[20] Servin B. Stephens M. 2007 Imputation-based analysis of association studies: candidate regions and quantitative traits. PLoS Genet. 3: e114. 17676998 10.1371/journal.pgen.0030114 PMC1934390

[21] Browning B. L. Browning S. R. 2009 A unified approach to genotype imputation and haplotype-phase inference for large data sets of trios and unrelated individuals. Am. J. Hum. Genet. 84: 210–223. 19200528 10.1016/j.ajhg.2009.01.005 PMC2668004

[22] Marchini J. Howie B. Myers S. McVean G. Donnelly P. 2007 A new multipoint method for genome-wide association studies by imputation of genotypes. Nat. Genet. 39: 906–913. 17572673 10.1038/ng2088

[23] Sitlani C. M. McKnight B. 2011 Relative efficiency of trend tests with misspecified genetic models in stratified analyses of case-control or cohort data. Hum. Hered. 71: 246–255. 21811075 10.1159/000328858 PMC3190174

[24] Skol A. D. Scott L. J. Abecasis G. R. Boehnke M. 2006 Joint analysis is more efficient than replication-based analysis for two-stage genome-wide association studies. *Nat. Genet.* 38: 209–213. 10.1038/ng1706 16415888

[25] Thompson S. G. Sharp S. J. 1999 Explaining heterogeneity in meta-analysis: a comparison of methods. Stat. Med. 18: 2693–2708. 10521860 10.1002/(sici)1097-0258(19991030)18:20<2693::aid-sim235>3.0.co;2-v

[26] Lemaitre R. N. Tanaka T. Tang W. Manichaikul A. Foy M. Kabagambe E. K. Nettleton J. A. King I. B. Weng L. C. Bhattacharya S. 2011 Genetic loci associated with plasma phospholipid n-3 fatty acids: a meta-analysis of genome-wide association studies from the CHARGE Consortium. PLoS Genet. 7: e1002193. 21829377 10.1371/journal.pgen.1002193 PMC3145614

[27] Hanada K. 2003 Serine palmitoyltransferase, a key enzyme of sphingolipid metabolism. Biochim. Biophys. Acta. 1632: 16–30. 12782147 10.1016/s1388-1981(03)00059-3

[28] Hicks A. A. Pramstaller P. P. Johansson A. Vitart V. Rudan I. Ugocsai P. Aulchenko Y. Franklin C. S. Liebisch G. Erdmann J. 2009 Genetic determinants of circulating sphingolipid concentrations in European populations. PLoS Genet. 5: e1000672. 19798445 10.1371/journal.pgen.1000672 PMC2745562

[29] Mullen T. D. Hannun Y. A. Obeid L. M. 2012 Ceramide synthases at the centre of sphingolipid metabolism and biology. Biochem. J. 441: 789–802. 22248339 10.1042/BJ20111626 PMC3689921

[30] Pewzner-Jung Y. Park H. Laviad E. L. Silva L. C. Lahiri S. Stiban J. Erez-Roman R. Brugger B. Sachsenheimer T. Wieland F. 2010 A critical role for ceramide synthase 2 in liver homeostasis: I. alterations in lipid metabolic pathways. J. Biol. Chem. 285: 10902–10910. 20110363 10.1074/jbc.M109.077594 PMC2856296

[31] Quehenberger O. Dennis E. A. 2011 The human plasma lipidome. N. Engl. J. Med. 365: 1812–1823. 22070478 10.1056/NEJMra1104901 PMC3412394

[32] Dougherty R. M. Galli C. Ferro-Luzzi A. Iacono J. M. 1987 Lipid and phospholipid fatty acid composition of plasma, red blood cells, and platelets and how they are affected by dietary lipids: a study of normal subjects from Italy, Finland, and the USA. Am. J. Clin. Nutr. 45: 443–455. 3812343 10.1093/ajcn/45.2.443

[33] Wu J. H. Lemaitre R. N. Manichaikul A. Guan W. Tanaka T. Foy M. Kabagambe E. K. Djousse L. Siscovick D. Fretts A. M. 2013 Genome-wide association study identifies novel loci associated with concentrations of four plasma phospholipid fatty acids in the de novo lipogenesis pathway: results from the Cohorts for Heart and Aging Research in Genomic Epidemiology (CHARGE) consortium. Circ. Cardiovasc. Genet. 6: 171–183. 23362303 10.1161/CIRCGENETICS.112.964619 PMC3891054

[34] Park T. S. Hu Y. Noh H. L. Drosatos K. Okajima K. Buchanan J. Tuinei J. Homma S. Jiang X. C. Abel E. D. 2008 Ceramide is a cardiotoxin in lipotoxic cardiomyopathy. J. Lipid Res. 49: 2101–2112. 18515784 10.1194/jlr.M800147-JLR200 PMC2533410

[35] Jiang X. C. Goldberg I. J. Park T. S. 2011 Sphingolipids and cardiovascular diseases: lipoprotein metabolism, atherosclerosis and cardiomyopathy. Adv. Exp. Med. Biol. 721: 19–39. 21910080 10.1007/978-1-4614-0650-1_2

[36] Deng X. Yin X. Allan R. Lu D. D. Maurer C. W. Haimovitz-Friedman A. Fuks Z. Shaham S. Kolesnick R. 2008 Ceramide biogenesis is required for radiation-induced apoptosis in the germ line of C. elegans. Science. 322: 110–115. 18832646 10.1126/science.1158111 PMC2585063

[37] Crowder C. M. 2009 Cell biology. Ceramides–friend or foe in hypoxia? Science. 324: 343–344. 19372418 10.1126/science.1173278 PMC3753222

[38] Lee S. Y. Kim J. R. Hu Y. Khan R. Kim S. J. Bharadwaj K. G. Davidson M. M. Choi C. S. Shin K. O. Lee Y. M. 2012 Cardiomyocyte specific deficiency of serine palmitoyltransferase subunit 2 reduces ceramide but leads to cardiac dysfunction. J. Biol. Chem. 287: 18429–18439. 22493506 10.1074/jbc.M111.296947 PMC3365730

[39] Rahmaniyan M. Curley R. W. Jr Obeid L. M. Hannun Y. A. Kraveka J. M. 2011 Identification of dihydroceramide desaturase as a direct in vitro target for fenretinide. J. Biol. Chem. 286: 24754–24764. 21543327 10.1074/jbc.M111.250779 PMC3137051

