# Supplementary material for: Genetic loci associated with circulating levels of very long-chain saturated fatty acids
Source: J Lipid Res. 2015 Jan;56(1):176–84. doi: 10.1194/jlr.M052456 (PMC4274065; doi:10.1194/jlr.M052456)
Supplement: Supplemental Data [file supp_56_1_176__index.html]

Genetic Loci Associated with Circulating Levels of Very Long-Chain Saturated Fatty Acids — Genetic loci associated with circulating levels of very long-chain saturated fatty acids — Supplemental Data 

# Genetic loci associated with circulating levels of very long-chain saturated fatty acids

## Supplemental Data

**Files in this Data Supplement:**

- Supplementary Text - Details of Participating Cohorts
- Supplementary Table 1 - Z-scores from the GWAS of 20:0, for rs2100944 and rs11666913 (chromosome 19), and for rs680379 (chromosome 20), in each study and the meta-analysis (overall)
- supplementary Table 2 - Meta-analysis results for the 250 most associated SNPs in the GWAS of 20:0
- supplementary Table 3 - Meta-analysis results for the 250 most associated SNPs in the GWAS of 22:0 adjusted for levels of 20:0
- supplementary Table 4 - Meta-analysis results for the 250 most associated SNPs in the GWAS of 24:0 adjusted for levels of 20:0
- supplementary Tables 5 and 6 - Supplementary Table 5. Estimated effect size (changes in 20:0 levels in SD units, per copy of the minor allele) from fixed effects meta-analyses, for rs2100944, rs11666913 and rs680379, stratified on plasma phospholipids vs erythrocytes and overall. Supplementary Table 6. Estimated effect size (changes in fatty acid levels in SD units, per copy of the minor allele) from fixed effects meta-analyses of the GWAS of 22:0 (A) and 24:0 (B) adjusted for 20:0, for rs2100944 and rs11666913, stratified on plasma phospholipids vs erythrocytes and overall.
- Supplementary figure 1 and supplementary figure 2 - Supplementary Figure 1. Manhattan Plot of GWAS of 22:0, without (A) and with (B) adjustment for 20:0 Supplementary Figure 2. Manhattan Plot of GWAS of 24:0, without (A) and with (B) adjustment for 20:0
