# Supplementary material for: Genetic loci associated with circulating levels of very long-chain saturated fatty acids
Source: J Lipid Res. 2015 Jan;56(1):176–84. doi: 10.1194/jlr.M052456 (PMC4274065; doi:10.1194/jlr.M052456)
Supplement: Supplemental Data [file supp_M052456_jlr.M052456-1.docx]

**Supplementary Text: Details of Participating Cohorts**

**Study Samples**

Participants (n=10,529) for the current meta-analysis of genome-wide association studies (GWAS) of long-chain saturated fatty acids were drawn from 7 cohorts, namely, the Atherosclerosis Risk in Communities Study (ARIC), the Coronary Artery Risk Development in Young Adults Study (CARDIA), the Cardiovascular Health Study (CHS), the Health Professionals Follow-up Study (HPFS), the Nurses’ Health Study (NHS), the Multi-Ethnic Study of Atherosclerosis (MESA) and the Women’s Genome Health Study (WGHS). These cohorts comprise the CHARGE (Cohorts for Heart and Aging Research in Genome Epidemiology) Consortium. All participants provided informed consent and the study protocols were approved by the local ethical committees at each participating institution.

**Study Samples, Phenotype, and Genotyping in the Participating Cohorts**

**The Atherosclerosis Risk in Communities Study**

The ARIC study is a multi-center prospective investigation of atherosclerotic disease in a predominantly bi-racial population ^1^. White and African American men and women aged 45- 64 years at baseline were recruited from 4 communities: Forsyth County, North Carolina; Jackson, Mississippi; suburban areas of Minneapolis, Minnesota; and Washington County, Maryland. A total of 15,792 individuals participated in the baseline examination in 1987-1989, with follow-up examinations in approximate 3-year intervals, during 1990-1992, 1993-1995, and 1996-1998.

ARIC Study samples were genotyped using the Affymetrix Genome-Wide Human SNP Array 6.0 (Santa Clara, California); for the current analysis only white participants were analyzed. Sample exclusion criteria included discordant with previous genotype data (n=83), genotypic and phenotypic sex mismatch (n=32), suspected first-degree relative of an included individual based on genotype data (n=297), genetic outlier as assessed by Identity by State (IBS) using PLINK ^2^ and >8 SD along any of the first 10 principal components in EIGENSTRAT ^3^ with 5 iterations (n=322). Autosomal SNPs were used for imputation after exclusion of SNPs with HWE deviation p<5 x 10^-5^, call rate <95%, or MAF<1%.

Fatty acids were measured in EDTA plasma that had been frozen at -70 ̊C. Fatty acid assays were performed at the Collaborative Studies Clinical Laboratory at Fairview-University Medical Center (Minneapolis, MN) as previously described ^4^. Lipids were extracted with chloroform/methanol and separated by thin layer chromatography. Fatty acid methyl esters were prepared from the phospholipid fraction and separated by gas chromatography using an HP-5890 gas chromatograph (Hewlett- Packard, Palo Alto, CA) with a 100-m capillary Varian CP7420 column. We identified 29 fatty acids. The concentration of each fatty acid was expressed as a percentage of total fatty acids.

**The Cardiovascular Health Study**

The CHS is a population-based longitudinal study of risk factors for cardiovascular disease and stroke in adults 65 years of age or older, recruited at four field centers (Forsyth County, NC; Sacramento County, CA; Washington County, MD; Pittsburgh, PA) ^5^. Overall, 5,201 predominantly Caucasian individuals were recruited in 1989-1990 from random samples of Medicare eligibility lists, followed by an additional 687 African-Americans recruited in 1992- 1993 (total n=5,888). The CHS GWAS, which had the primary aim of studying incident cardiovascular events, focused on 3,980 participants who were free of clinical cardiovascular disease at study baseline, consented to genetic testing, and had DNA available for genotyping. A total of 1,908 persons were excluded from the GWAS study sample due to the presence at study baseline of coronary heart disease, congestive heart failure, peripheral vascular disease, valvular heart disease, stroke, or transient ischemic attack. Fatty acids were measured on samples collected in the 3^rd^ year of follow-up.

CHS Study samples were genotyped using the Illumina HumanCNV370-Duo BeadChip system. Because the other cohorts were predominantly of European descent, the African American participants were excluded from this analysis. Genotyping was successful in 3,291 Caucasian subjects. Participants were eligible for the present investigation if their genotyping was complete and they had available phenotype information. Samples with call rate <95% were excluded. A total of 306,655 autosomal SNPs were used in imputation after filtering out SNPs with HWE deviation p≤1 x 10^-5^, call frequency ≤97%, zero heterozygote frequency, missing from dbSNP, and >1 duplicate or Mendelian inconsistency.

Blood was drawn after a 12-hour fast and stored at -70^o^C. Measurements were performed at the Fred Hutchinson Cancer Research Center, providing quantitative measurement of 42 fatty acids. Total lipids were extracted from plasma using methods of Folch ^6^, and phospholipids separated from neutral lipids by one-dimensional TLC. Fatty-acid-methyl-ester (FAME) samples were prepared by direct transesterification using methods of Lepage and Roy ^7^, and separated using gas chromatography (Agilent5890 gas- chromatograph-FID-detector; Supelco fused-silica 100m capillary column SP-2560; initial 160°C 16 min, ramp 3.0°C/min to 240°C, hold 15 min) ^8^. Identification, precision, and accuracy were continuously evaluated using model mixtures of known FAMEs and established in-house controls, with identification confirmed by GC-MS at USDA (Peoria, IL). CVs were <3% for most fatty acids.

**The Coronary Artery Risk Development in Young Adults Study**

The CARDIA Study is a prospective multicenter study with 5,115 adults Caucasian and African American participants of the age group 18-30 years, recruited from four centers. The recruitment was done from the total community in Birmingham, AL, from selected census tracts in Chicago, IL and Minneapolis, MN; and from the Kaiser Permanente health plan membership in Oakland, CA. The details of the study design for the CARDIA study have been published before ^9^. Eight examinations have been completed since initiation of the study in 1985–1986, respectively, in the years 0, 2, 5, 7, 10, 15, 20 and 25. Written informed consent was obtained from participants at each examination and all study protocols were approved by the institutional review boards of the participating institutions.

CARDIA Study samples from were genotyped using the Affymetrix Genome-Wide Human SNP Array 6.0 (Santa Clara, California); only participants of European descent were included in the GWAS analyses. Genotyping was completed for 1,720 individuals with a sample call rate ≥ 98%. A total of 578,568 SNPs passed quality control (MAF ≥ 2%, call rate ≥ 95%, HWE ≥ 10^-4^) and were used for imputation. For this study, complete genotype and phenotype information were available for 1,507 individuals.

Fatty acids were measured in EDTA plasma, frozen at –70°C, using methods previously described by Cao et al ^4^. Lipids are extracted from the plasma using a chloroform/methanol extraction method and the cholesterol esters, triglyceride, phospholipids and free fatty acids are separated by thin layer chromatography. The fatty acid methyl esters are obtained from the phospholipids and are detected by gas chromatography flame ionization. Individual fatty acids are expressed as a percent of total fatty acids. 28 fatty acids were identified.

**The Multi-Ethnic Study of Atherosclerosis**

The MESA Study is a study of the characteristics of subclinical cardiovascular disease (disease detected non-invasively before it has produced clinical signs and symptoms) and the risk factors that predict progression to clinically overt cardiovascular disease or progression of the subclinical disease ^10^. MESA researchers study a diverse, population-based sample of 6,814 asymptomatic men and women aged 45-84. Thirty-eight percent of the recruited participants are white, 28 % African-American, 22 % Hispanic, and 12 % Asian, predominantly of Chinese descent. Participants were recruited from six field centers across the United States: Wake Forest University, Columbia University, Johns Hopkins University, University of Minnesota, Northwestern University and University of California - Los Angeles.

MESA samples were genotyped using the Affymetrix Genome-Wide Human SNP Array 6.0 (Santa Clara, California); for the current meta-analysis only self-reported Caucasian participants were analyzed, while MESA Chinese, African American and Hispanic samples are included in the look-up of top SNPs. Sample exclusion criteria included heterozygosity > 53% and individual-level genotyping call rate < 95%. Monomorphic SNPs were removed, and there was no filter on HWE or MAF. IMPUTE version 2.1.0 was used to perform imputation for the MESA SHARe Caucasian participants (chromosomes 1-22) using HapMap Phase I and II - CEU as the reference panel (release #24 - NCBI Build 36 (dbSNP b126)). Relationship inference was performed using KING ^11^ to identify first- and second- degree relatives, and an unrelated set of individuals was identified for genome-wide association analysis.

Fatty acids were obtained for a subset of 2,767 individuals with genotypes available through MESA SHARe, with approximately equal representation from the four ethnic groups (713 Caucasians, 712 Chinese, 645 African Americans, and 697 Hispanics). The fatty acids were measured in EDTA plasma, frozen at –70°C, using methods previously described by Cao et al ^4^ and detailed above for the ARIC study.

**The Health Professionals Follow-up Study (HPFS) and Nurses’ Health Study (NHS)**

The HPFS began in 1986 when 51,529 U.S. health professionals (dentists, optometrists, pharmacists, podiatrists, and veterinarians), aged 40 to 75 years, answered a detailed questionnaire that included a comprehensive diet survey, and items on lifestyle practice and medical history. The NHS consisted of 121,700 registered female nurses, age 30 to 55 years, who lived in one of 11 states and completed a baseline questionnaire about their lifestyle and medical history in 1976. In both cohorts, follow-up questionnaires have been administered biennially to update information on exposures and newly diagnosed diseases. In 1980, 1984, 1986 and every 4 years thereafter, a validated food frequency questionnaire has been sent to NHS participants to collect and update information on diet, alcohol, and vitamin supplements. In the HPFS the same questionnaire has been sent to HPFS participants every 4 years. In 1993-1995, blood samples were obtained from 18,224 HPFS participants, and between 1989 and 1990, blood samples were collected from 32,826 women. Upon arrival whole blood samples were centrifuged and stored in cryotubes as plasma, buffy coat, and red blood cells in the vapor phase of liquid nitrogen freezers. DNA was extracted from the buffy coat fraction of centrifuged blood with the QLAmp Blood Kit (Qiagen, Chatsworth, California).

A nested case-control study of coronary heart disease (CHD; including non-fatal myocardial infarction and fatal CHD events) was conducted among participants who provided blood samples in both cohorts. Self-reported incidence of CHD events was confirmed by exposure-blinded study physicians through reviewing medical records. Participants with existing cardiovascular disease or cancer at blood draw were excluded from the case-control study. Controls were selected randomly using risk-set sampling and matched to cases in a 2:1 ratio on age, smoking, and month of blood return; in the NHS fasting status was further matched between cases and controls. Erythrocyte fatty acid concentrations were determined by gas-liquid chromatography among 1,334 CHD cases and controls in the HPFS and 1131 case-control triplets in the NHS as described previously ^12^. Briefly, fatty acids in erythrocytes were first extracted into isopropanol and hexane and then transmethylated with methanol and sulfuric acid. Fatty acid methyl esters were evaporated and re-dissolved in isooctane and then measured by gas-liquid chromatography. Individual peaks were identified by comparison with known standards, and each peak was quantified by calculating the area under the peak. The concentration of each individual fatty acid was expressed as a percentage of total area under the peaks.

Genotyping was performed using the Affymetrix SNP 6.0 array and the Birdseed calling algorithm. A total of 1330 HPFS samples (98%) passed laboratory technical quality control criteria, and 96% of NHS samples passed quality control. Population structure was determined using principal component analysis. A set of 12,021 SNPs with very low levels of linkage disequilibrium and minor allele frequency >0.05 in Caucasians were selected and used to construct the principal components of ethnicity ^13^. Study participants passing quality control were analyzed together with a set of 209 HapMap II founders (59 CEU, 60 YRI, 45 JPT and 45 CHB). Participants within the means of the first and second principal components (mean (SD) = 3) among self-described Caucasians were classified as having primarily European ancestry. We excluded participants with substantial evidence of non-European genetic ancestry from subsequent analysis. In addition, SNPs that were monomorphic, had call rate <98%, a Hardy-Weinberg Equilibrium p-value <1×10^-4^, or a minor allele frequency <0.02 were excluded, leaving a total of 724,881 SNPs in the HPFS or 721,316 in the NHS that passed quality control analysis of called genotypes. Imputation of ~2.5 million SNPs was performed using MACH software (v1.0.16) with HapMap CEU phased II data (Release 22) as the reference panel.

**The Women’s Genome Health Study**

The Women’s Health Study (WHS) is a randomized trial evaluating the risks and benefits of low-dose aspirin and vitamin E in primary prevention of cardiovascular disease and cancer among 39,876 US women ^14, 15^. A third component, β-carotene, was initially included in the trial but terminated after a median treatment of 2.1 y ^16^. From 1992 to 1995, 39,876 US female health professionals, aged ≥39 years and free from cardiovascular disease and cancer (except non-melanoma skin cancer), were randomized into the WHS. Baseline blood samples were collected from 28,345 participants and stored in liquid nitrogen freezers.

The Women’s Genome Health Study is the subset of WHS participants of European ancestry with complete genome-wide genotype data ^17^. Genotyping was performed using the Illumina’s Infinium II assay ^18^ applied to the HumanHap300 Duo ‘+’ platform, including a genome-wide set of haplotype-tagging SNP markers suitable for populations with European ancestry and custom content to enhance coverage of genomic regions of significance in cardiovascular disease ^19^. In the experimental data, all samples were required to have successful genotyping for at least 98% of the SNPs, SNPs were retained with minor allele frequency (MAF) >1%, successful genotyping in at least 90% of the subjects, and deviations from Hardy-Weinberg equilibrium using an exact test not exceeding P=1.0×10^-6^ in significance. Finally, a subset of 23,294 participants of self-reported European ancestry verified by a multidimensional scaling procedure in PLINK (<http://pngu.mgh.harvard.edu/purcell/plink>) had 339,000 genotyped SNPs remained in the final data after applying quality control filters, and up to a total of 2.6 million SNPs were imputed with MaCH v. 1.0.16 (http://www.sph.umich.edu/csg/abecasis/mach/) using the reference panel from the HapMap2 CEU population ^20^.

The fatty acid (FA) profile in erythrocyte membranes was measured in WHS participants at the Department of Laboratory Medicine and Pathology, University of Minnesota, using the method by Cao *et al* ^4^*.* FAs were extracted with a mixture of chloroform and methanol (2:1, v/v), dissolved in heptane, and injected onto a capillary Varian CP7420 100-m column with a Hewlett Packard 5890 gas chromatograph (GC) equipped with a HP6890A autosampler. The GC was configured for a single capillary column with a flame ionization detector and interfaced with HP chemstation software. Adequate separation of FA methyl esters was obtained over a 50-min period with an initial temperature of 190ºC followed by subsequent temperature gradually increased to 240ºC. A total of 28 FAs from 14:0 through 24:1(n-9) were separated, identified and expressed as percent of total FA.

**References**

1. The atherosclerosis risk in communities (aric) study: Design and objectives. The aric investigators. Am J Epidemiol 1989;129:687-702.

2. Purcell S, Neale B, Todd-Brown K, et al.: Plink: A tool set for whole-genome association and population-based linkage analyses. Am J Hum Genet 2007;81:559-575.

3. Price AL, Patterson NJ, Plenge RM, Weinblatt ME, Shadick NA, Reich D: Principal components analysis corrects for stratification in genome-wide association studies. Nat Genet 2006;38:904-909.

4. Cao J, Schwichtenberg KA, Hanson NQ, Tsai MY: Incorporation and clearance of omega-3 fatty acids in erythrocyte membranes and plasma phospholipids. Clin Chem 2006;52:2265-2272.

5. Fried LP, Borhani NO, Enright P, et al.: The cardiovascular health study: Design and rationale. Ann Epidemiol 1991;1:263-276.

6. Folch J, Lees M, Sloane GH: A simple method for isolation and purification of total lipids from animal tissues. The Journal of biological chemistry 1957;226:497-509.

7. Lepage G, Roy CC: Direct transesterification of all lipids in a one-step reaction. J Lipid Res 1986;27:114-120.

8. Mozaffarian D, Cao H, King IB, et al.: Circulating palmitoleic acid and risk of metabolic abnormalities and new-onset diabetes. The American journal of clinical nutrition 2010;92:1350-1358.

9. Friedman GD, Cutter GR, Donahue RP, et al.: Cardia: Study design, recruitment, and some characteristics of the examined subjects. J Clin Epidemiol 1988;41:1105-1116.

10. Bild DE, Bluemke DA, Burke GL, et al.: Multi-ethnic study of atherosclerosis: Objectives and design. Am J Epidemiol 2002;156:871-881.

11. Manichaikul A, Mychaleckyj JC, Rich SS, Daly K, Sale M, Chen WM: Robust relationship inference in genome-wide association studies. Bioinformatics 2010;26:2867-2873.

12. Baylin A, Kim MK, Donovan-Palmer A, et al.: Fasting whole blood as a biomarker of essential fatty acid intake in epidemiologic studies: Comparison with adipose tissue and plasma. Am J Epidemiol 2005;162:373-381.

13. Yu K, Wang Z, Li Q, et al.: Population substructure and control selection in genome-wide association studies. PLoS One 2008;3:e2551.

14. Cook NR, Lee IM, Gaziano JM, et al.: Low-dose aspirin in the primary prevention of cancer: The women's health study: A randomized controlled trial. JAMA : the journal of the American Medical Association 2005;294:47-55.

15. Lee IM, Cook NR, Gaziano JM, et al.: Vitamin e in the primary prevention of cardiovascular disease and cancer: The women's health study: A randomized controlled trial. JAMA : the journal of the American Medical Association 2005;294:56-65.

16. Lee IM, Cook NR, Manson JE, Buring JE, Hennekens CH: Beta-carotene supplementation and incidence of cancer and cardiovascular disease: The women's health study. J Natl Cancer Inst 1999;91:2102-2106.

17. Ridker PM, Chasman DI, Zee RY, et al.: Rationale, design, and methodology of the women's genome health study: A genome-wide association study of more than 25,000 initially healthy american women. Clin Chem 2008;54:249-255.

18. Gunderson KL, Steemers FJ, Ren H, et al.: Whole-genome genotyping. Methods Enzymol 2006;410:359-376.

19. Gunderson KL, Kuhn KM, Steemers FJ, Ng P, Murray SS, Shen R: Whole-genome genotyping of haplotype tag single nucleotide polymorphisms. Pharmacogenomics 2006;7:641-648.

20. International HapMap C, Frazer KA, Ballinger DG, et al.: A second generation human haplotype map of over 3.1 million snps. Nature 2007;449:851-861.
