# Supplementary material for: Genetic loci associated with circulating levels of very long-chain saturated fatty acids
Source: J Lipid Res. 2015 Jan;56(1):176–84. doi: 10.1194/jlr.M052456 (PMC4274065; doi:10.1194/jlr.M052456)
Supplement: Supplemental Data [file supp_M052456_jlr.M052456-2.docx]

Supplementary Table 1. Z-scores from the GWAS of 20:0, for rs2100944 and rs11666913 (chromosome 19), and for rs680379 (chromosome 20), in each study and the meta-analysis (overall)

| Study | rs2100944 | rs11666913 | rs680379 |
| --- | --- | --- | --- |
| ARIC | -7.51 | 5.51 | 3.62 |
| CARDIA | NA | NA | 1.21 |
| CHS | -10.92 | 9.08 | 5.20 |
| HPFS | -4.24 | 3.70 | 3.38 |
| MESA | NA | NA | 1.02 |
| NHS | -3.50 | 1.60 | 1.42 |
| WGHS | -0.64 | 1.68 | 1.87 |
| OVERALL | -13.29 | 11.04 | 7.21 |
