# Supplementary material for: Genetic loci associated with circulating levels of very long-chain saturated fatty acids
Source: J Lipid Res. 2015 Jan;56(1):176–84. doi: 10.1194/jlr.M052456 (PMC4274065; doi:10.1194/jlr.M052456)
Supplement: Supplemental Data [file supp_M052456_jlr.M052456-6.docx]

MS ID#: JLR/2014/052456

MS TITLE: Genetic Loci Associated with Circulating Levels of Very Long-Chain

Saturated Fatty Acids

**Supplementary Table 5.** Estimated effect size (changes in 20:0 levels in SD units, per copy of the minor allele) from fixed effects meta-analyses, for rs2100944, rs11666913 and rs680379, stratified on whether 20:0 was measured in plasma phospholipids or erythrocytes, and overall.

|  | rs2100944 | rs11666913 | rs680379 |
| --- | --- | --- | --- |
| Plasma phospholipids | -0.347 (-0.398, -0.295) | 0.285 (0.232, 0.337) | 0.098 (0.066, 0.130) |
| Erythrocytes | -0.216 (-0.306, -0.125) | 0.179 (0.092, 0.267) | 0.133 (0.069, 0.197) |
| OVERALL | -0.314 (-0.359, -0.269) | 0.257 (0.212, 0.302) | 0.105 (0.077, 0.134) |
| P value* | 0.01 | 0.04 | 0.34 |

* p value for test of the difference between VLSFA measured in plasma phospholipids or erythrocytes.

**Supplementary Table 6.** Estimated effect size (changes in fatty acid levels in SD units, per copy of the minor allele) from fixed effects meta-analyses of the GWAS of 22:0 (A) and 24:0 (B) adjusted for 20:0, for rs2100944 and rs11666913, stratified on whether 22:0/24:0 were measured in plasma or erythrocyte phospholipids, and overall.

**A) 22:0**

|  | rs2100944 | rs11666913 |
| --- | --- | --- |
| Plasma phospholipids | 0.189 (0.156, 0.222) | -0.165 (-0.200, -0.130) |
| Erythrocytes | 0.089 (0.013, 0.165) | -0.032 (-0.107, 0.043) |
| OVERALL | 0.173 (0.143, 0.204) | -0.141 (-0.173, -0.110) |
| P value* | 0.02 | 0.002 |

**B) 24:0**

|  | rs2100944 | rs11666913 |
| --- | --- | --- |
| Plasma phospholipids | 0.234 (0.192, 0.276) | -0.208 (-0.249, -0.167) |
| Erythrocytes | 0.007 (-0.073, 0.088) | -0.023 (-0.102, 0.055) |
| OVERALL | 0.186 (0.149, 0.223) | -0.168 (-0.204, -0.131) |
| P value* | <0.001 | <0.001 |

* p value for test of the difference between VLSFA measured in plasma phospholipids or erythrocytes.
