# Supplementary material for: Genetic loci associated with circulating levels of very long-chain saturated fatty acids
Source: J Lipid Res. 2015 Jan;56(1):176–84. doi: 10.1194/jlr.M052456 (PMC4274065; doi:10.1194/jlr.M052456)

Supplementary Figure 1. Manhattan Plot of GWAS of 22:0, without (A) and with (B) adjustment for 20:0

A B


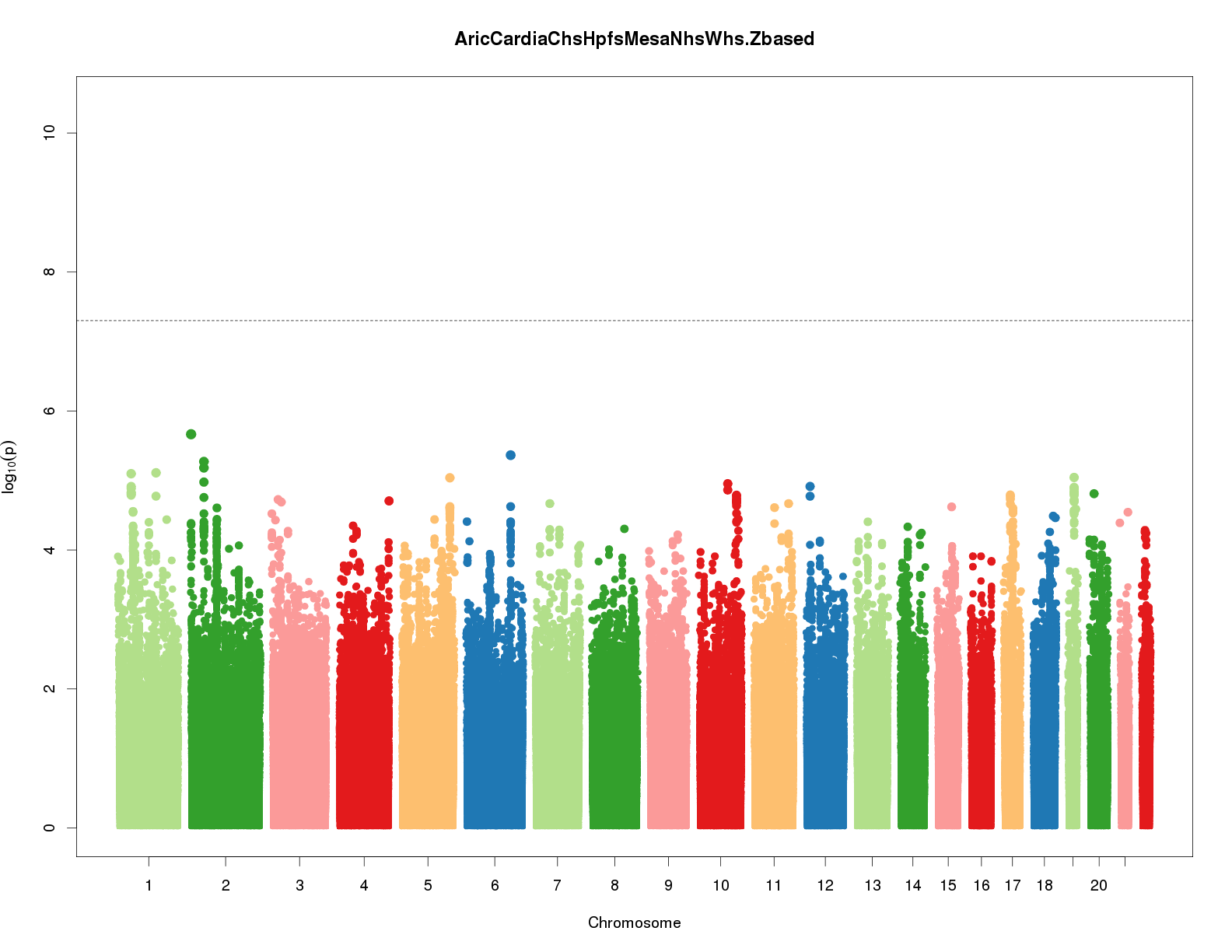

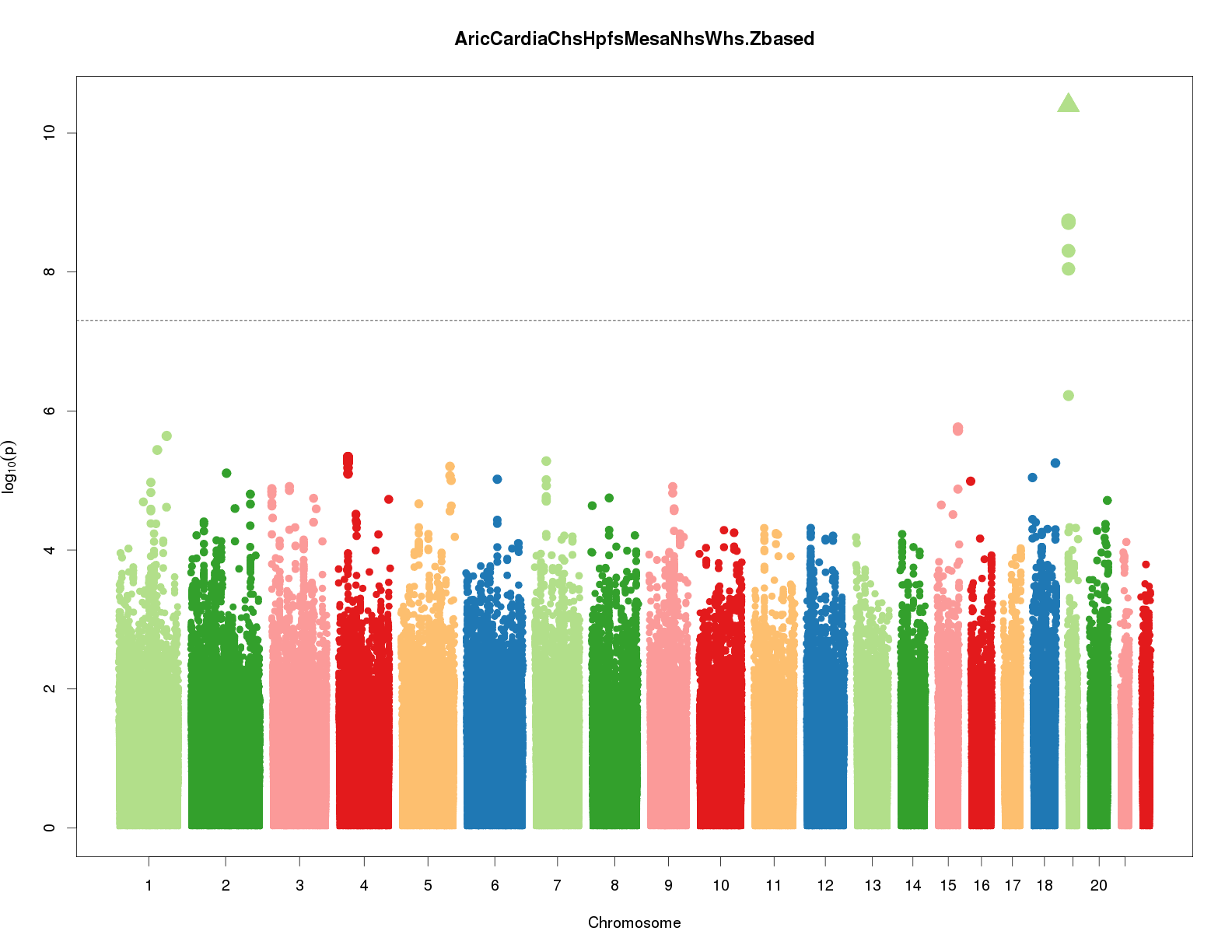


Supplementary Figure 2. Manhattan Plot of GWAS of 24:0, without (A) and with (B) adjustment for 20:0

A B


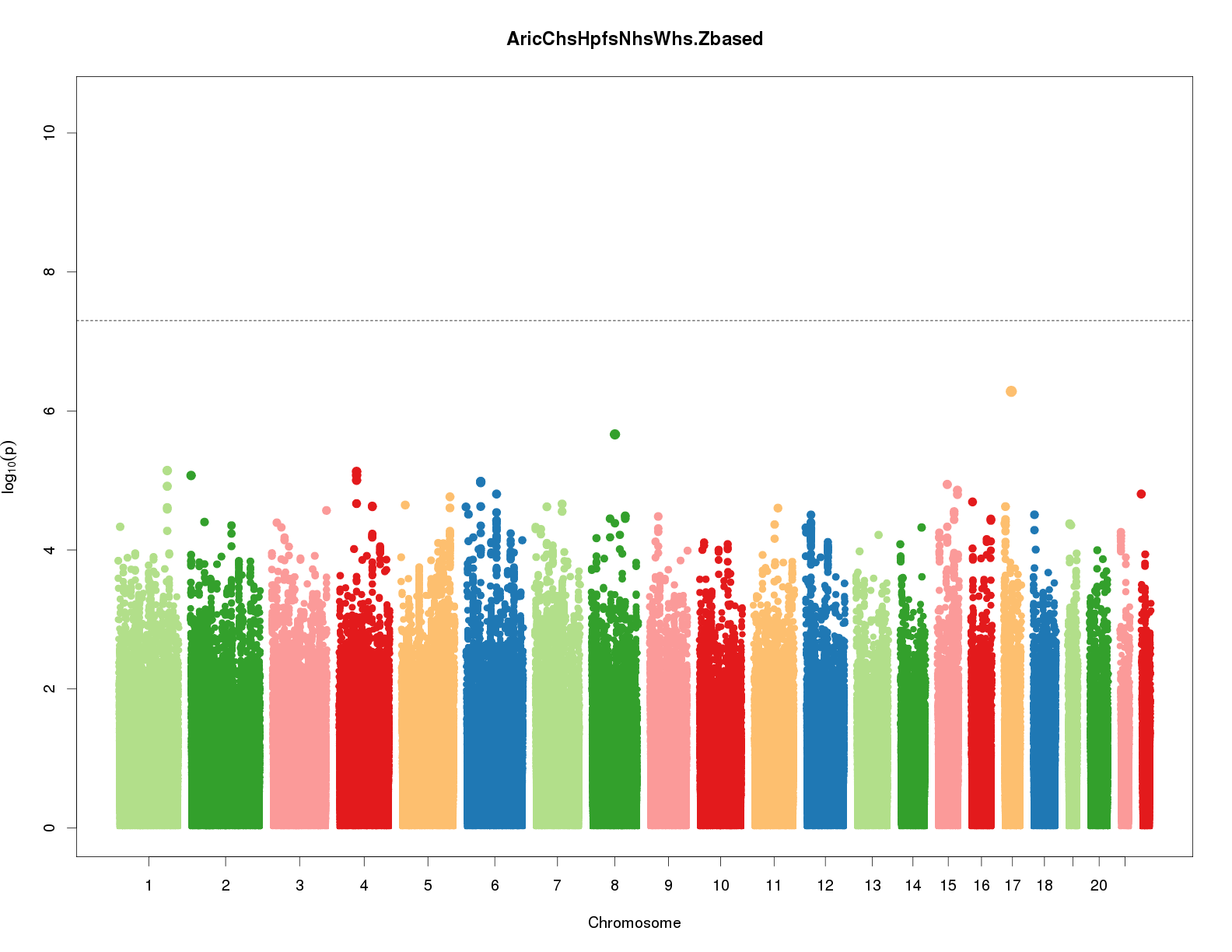

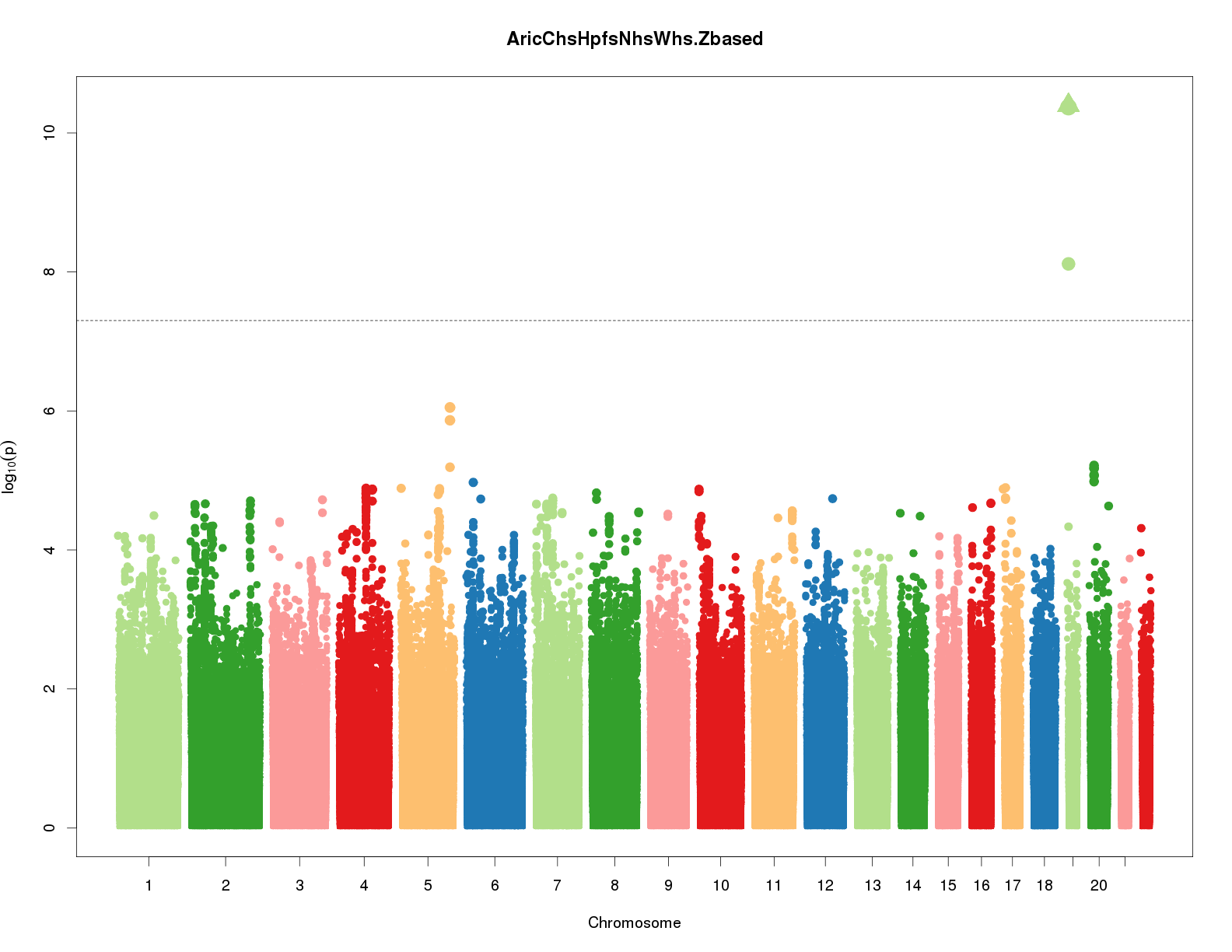

Supplement: Supplemental Data [file supp_M052456_jlr.M052456-7.docx]
